# The rs35874116 single nucleotide polymorphism increases sweet intake and the risk of severe early childhood caries: a case–control study

**DOI:** 10.1186/s12903-022-02512-8

**Published:** 2022-11-05

**Authors:** Yan Liang, Junyu Yao, Rongmin Qiu, Aihua Chen, Hua Huang, Huancai Lin, Lixia Yu

**Affiliations:** 1 College of Stomatology, Hospital of Stomatology, Guangxi Medical University, Guangxi Key Laboratory of Oral and Maxillofacial Rehabilitation and Reconstruction, Guangxi Clinical Research Center for Craniofacial Deformity, Guangxi Key Laboratory of Oral and Maxillofacial Surgery Disease Treatment, Guangxi Health Commission Key Laboratory of Prevention and Treatment for Oral Infectious Diseases, Nanning, Guangxi 530021 China; 2 Department of Pediatric Dentistry, Affiliated Stomatology Hospital of Guilin Medical University, Guilin, 541001 China; 3 Department of Preventive Dentistry, Guanghua School of Stomatology, Hospital of Stomatology, Sun Yat-sen University, Guangdong Provincial Key Laboratory of Stomatology, Guangzhou, 510055 China

**Keywords:** Single nucleotide polymorphism, Sweet receptor gene, Sweet intake, Severe early childhood caries

## Abstract

**Background:**

Dental caries is one of the most prevalent chronic diseases worldwide among children. The rs35874116 single nucleotide polymorphism (SNP) in the sweet receptor gene *TAS1R2* has been associated with dental caries at a high risk in permanent teeth among school children and adults. To date, little is known about the association of this SNP with sweet intake and caries risk in the primary school children.

**Methods:**

Total of 236 children were included, namely 118 subjects in the non-caries (NC) group and severe early childhood caries (S-ECC) group, respectively. Oral mucosa cells were collected from all the selected children, and the full length of exon 3 in *TAS1R2* was sequenced to analyse rs35874116 polymorphism. A questionnaire was used to collect information about socio-demographic information, frequency of sweet intake and oral hygiene habits. Multiple logistic regression models were applied to assess the relationship of rs35874116 polymorphism with frequency of sweet intake and S-ECC among the five-year-old children.

**Results:**

Children with the TT genotype of rs35874116 had a higher frequency of sweet intake than CT/CC carriers (51.3% vs. 32.7%; *x*^2^ = 5.436, *p* = 0.020), and S-ECC individuals were more likely to be TT genotype carriers than NC individuals (53.5% vs. 46.5%; *x*^2^ = 4.353, *p* = 0.037). Multiple logistic regression analyses showed that the TT genotype of rs35874116 was not only significantly related to the frequency of sweet intake (OR = 2.25, 95% CI = 1.14–4.44) but also significantly associated with S-ECC (OR = 2.11, 95% CI = 1.01 ~ 4.42).

**Conclusions:**

The rs35874116 polymorphism might increase sweet intake and the risk of S-ECC among five-year-old children in Nanning, China.

## Background

Dental caries is one of the most prevalent chronic diseases worldwide among children. It is reported that 70.9% of five-year-old Chinese children suffer from dental caries [1]. It has been revealed that the amount and frequency of sugar intake are the most important dietary factors in caries development [2]. Furthermore, increasing sugar consumption is associated with a higher prevalence of caries. Therefore, determining the impact factors related to human sweet intake can help to promote caries prevention.

Sweet preferences are shaped by a high number of environmental, cultural, and nutritional factors, including genetic. Genetic variation plays a crucial role in individual differences in sweet preferences, which ultimately influence selection of dietary intake and health [3]. The relationship between genetic variation and taste has previously been investigated by examining single nucleotide polymorphisms (SNPs) in taste receptor genes with measures of taste sensitivity, taste preference, and dietary intake [4, 5].

Taste receptor family 1 members (T1Rs) lead to the perception of sweet and umami taste [6, 7]. The variation of the taste receptor T1R2 encoded by gene *TAS1R2* is related to interindividual differences in sugar sensitivity. To demonstrate the relationship between the *TAS1R2* gene and sweet intake as well as sugar-dependent caries disease, studies have focused on the relationship between rs35874116 polymorphism in the *TAS1R2* gene with sweet consumption and dental caries over the past few years [8–17].

The rs35874116 polymorphism was classified into three genotypes based on the mutational alleles: 1) homozygous polymorphic genotype TT, 2) homozygous wild-type CC, and 3) heterozygous type CT. The polymorphism rs35874116 is the substitution of an isoleucine for valine at position 191. TT indicates those that are homozygous valine/valine at position 191, and CT and CC indicates who are isoleucine carriers (either valine/isoleucine or isoleucine/isoleucine) at position 191. It was shown that people who are TT type carrier have greater sugar consumption [8, 15]. However, such an association was not found in some studies [10, 13, 16, 17]. A significant association was shown between total caries experience and the rs35874116 SNP, in which the TT genotype seemed to indicate a high risk for dental caries in permanent teeth among school children and adults [9, 11, 17], but there was no relation between variations in rs35874116 and caries of the primary dentition [11]. To date, little is known about whether this SNP was associated with sweet intake and the risk of caries in primary teeth.

In this study, we targeted rs35874116 polymorphism and explored the association of rs35874116 polymorphism with sweet intake and caries risk in five-year-old Chinese children. Our hypothesis was that rs35874116 polymorphism might be associated with the child’s frequency of sweet intake and severe childhood caries (S-ECC).

## Materials and methods

### Study subjects and sample size

A case-control group–design was applied in this study. The control group was the non-caries (NC) group, and the case group was S-ECC group. The criteria for the S-ECC group were those children with decayed, missing, and filled teeth (dmft) value equal to or more than six [18], and the non-caries (NC) group had no caries. The sample size for this study was determined based on the formulas of case-control study [19] as the followings:

1 $$N=\frac{N^{\prime }}{4}{\left(1+\sqrt{1+\frac{4}{N^{\prime}\delta }}\right)}^2$$

2 $${N}^{\prime }=\frac{{\left[{Z}_{\alpha}\sqrt{\left(1+{}^{1}\!\left/ \!{}_{C}\right.\right){\pi}_C\left(1-{\pi}_C\right)}+{Z}_{\beta}\sqrt{\pi_2\left(1-{\pi}_2\right)+{\pi}_1\left(1-{\pi}_1\right)/C}\right]}^2}{{\left({\pi}_2-{\pi}_1\right)}^2}$$

3 $${\pi}_C=\frac{\pi_2+{\pi}_1}{2}$$

4 $$\delta =\left|{\pi}_1-{\pi}_2\right|$$

The number of children was set to be equal in both groups. Thus, the value of *C* was 1.0. The value of *α* and *β* were set at 0.05 and 015, respectively. Z_α_ = 1.282 and Z_β_ = 1.96. The values of *π*_*1*_ and *π*_*2*_ referred to the predicted mutation rates of rs35874116 in the control and case groups. The largest sample size was required when *π*_*1*_ = 0.8 and *π*_*2*_ = 0.6, respectively. Therefore, the calculated sample size should be 118 children for each group. All statistical tests were two-sided.

The subjects were recruited from our previous study [20]. In total, 433 five-year-old children who had no systematic disease were randomly recruited from five kindergartens, Nanning, Guangxi, China, from September to December 2016. The clinical examination was carried out for all the children, and the details was described later. Among these 433 children, 155 of them had no caries, and 278 of them had caries. Base on calculated sample size, 118 of the children without caries was randomly recruited for NC group, and 118 subjects with dmft ≥6 was randomly recruited for S-ECC group. Therefore, total of 236 children were included.

Before the investigation, this study was approved by the Medical Ethics Committee of Guangxi Medical University (No.2015-2-12-1) and written informed consent for participation was obtained from the caregivers. All methods were performed in accordance with the Declaration of Helsinki.

### Data collection

A questionnaire designed based on our previous study [21] was used to collect socio-demographic information about the children (e.g., gender, age, single child, type of caregiver, parents’ marital status, educational level, and occupation, monthly family income), frequency of sweet intake and oral hygiene habits which included frequency of teeth brushing and use of fluoride toothpaste. The frequency of sweet intake was measured by one question (how often does your child have sugary snack per day), and there were two answers (< once/day and ≥ once/day). Children who had a sweet intake < once/day were treated as the low frequency of sweet intake group (LS group) and those with a sweet intake ≥ once/day were treated as the high frequency of sweet intake group (HS group). The frequency of teeth brushing was also measured by one question (how often does your child brushes his/her teeth daily), and there were two answers (≤once/day and ≥ twice/day). In this study, the combination of socio-demographic factors, frequency of sweet intake and oral hygiene habits were defined as social behaviour factors.

The questionnaire was pilot tested among the caregivers of the 5-year-old children prior to the study. In the study, the questionnaires were distributed by the kindergartens to the caregivers, who completed the questionnaire at home and returned it to the kindergartens.

### Clinical examination

Clinical examinations were performed in the classroom under natural light with the children lying on a desk and the examiner seated on a chair behind the subject. The examiner was trained and calibrated for dental caries diagnosis based on the World Health Organization (WHO) Health Survey Methods for field studies [22]. Caries status was recorded using dmft index for primary teeth. An intra-examiner calibration was performed weekly. The intra-examiner kappa values for dmft scores were > 0.89.

### Sample collection and SNP genotyping

Fasting was requested 30 minutes before oral mucosa cell collection for all the children. A disposable oral mucosa swab was used to non-invasively scrape the buccal mucosa back and forth approximately 30 times to get enough oral mucosa cells, which required full contact with oral mucosa, taking care to not touch the degreased cotton part. The wiped swab was put into the collection tube, which was sealed and kept in a foam box with dry ice and then sent to the lab within 4 h. All the swabs were marked and stored at − 20 °C and sent for genotyping within a week.

Extraction of DNA from mucosal cells was carried out using a DNA Extraction Kit (Tsingke). DNA was successfully extracted and identified as purity by 1% agarose gel electrophoresis and stored at − 20 °C. Exon 3 is located at 4676 bp–5449 bp, with a length of 774 bp. A pair of primers was designed according to the corresponding intron to amplify the full length of exon 3 (product full length 990 bp):

- Tas-exon3-F1: 5′- GGTGGCTTGCACTAGGTG-3′;
- Tas-exon3-R1:5 ‘- GTGTCTGGAAGAATGGGATA-3’.

PCR amplification products were detected by 2% agarose gel electrophoresis. Reaction system (50 μl): 2 x TSINGKE Master Mix (blue) 25 μl, TAS-EXON3-F1 (10p) 2 μl, Tas-exon3-R1 (10p) 2 μl, genomic DNA template 4 μl, dd H_2_O 17 μl. The reaction conditions were as follows: initial denaturation at 96 °C for 5 min; 35 cycles of denaturation at 96 °C for 30 s, primer annealing at 54 °C for 30 s, and extension at 72 °C for 1 min; and a final extension at 72 °C for 5 min. Amplification products were stored at 4 °C. A DNA gel magnetic beads recovery kit (Tsingke) was used to purify the target fragments. The purified target products were used for full-length bidirectional gene sequencing by the Guangzhou Tsingke Biotechnology Company (Guangzhou, China). Variant Reporter software was used to analyse the sequencing results, and rs35874116 sequence was selected as a reference sequence. The rs35874116 polymorphism was classified into three genotypes (genotype TT, CT and CC).

### Statistical analysis

SPSS 22.0 was used for statistical analysis. A chi-square test was used to compare the distributions of rs35874116 polymorphism (TT type vs. CT / CC type) between different groups of sweet intake frequency ((LS group and HS group) and between different groups caries statuses (NC group and S-ECC group). Multiple logistic regression analyses (back stepwise: conditional) were conducted to analyse potential relationship between rs35874116 polymorphism and the frequency of sweet intake or caries risk, controlling demographic and other potential confounding variables. A *p* value of less than 0.05 for all two-sided statistical tests was considered significant.

## Results

### Characteristics of the subjects

The distribution of social demographic factors between the LS and HS groups, and, between the NC and S-ECC groups are shown in Tables 1 and 2, respectively.

**Table 1:** Distribution of socio-demographic factors between LS and HS group^a^

Indicator	LS group (*n* = 124)	HS group (*n* = 112)	*x*^2^	*P*
Gender			1.665	0.241
Boy	56 (45.2)	60 (53.6)		
Girl	68 (54.8)	52 (46.4)		
Single child			0.31	0.601
No	52 (41.9)	51 (45.5)		
Yes	72 (58.1)	61 (54.5)		
Caregiver’s type			2.276	0.471
Parent	95 (76.6)	83 (74.1)		
Grandparent	29 (23.4)	27 (24.1)		
Other	0 (0.00)	2 (1.80)		
Marital status of parents			3.675^b^	0.124
Cohabiting	120 (96.8)	112 (100)		
Not Cohabiting	4 (3.20)	0 (0.00)		
Mother’s education			0.476	0.559
≤ 12 year	14 (11.3)	16 (14.3)		
>12 year	110 (88.7)	96 (85.7)		
Father’s education			0.057	0.820
≤ 12 year	10 (8.1)	10 (8.9)		
>12 year	114 (91.9)	102 (91.1)		
Mother’s occupation			0.892^b^	0.753
Employer / Professional	82 (66.1)	70 (62.5)		
Employee / Non-professional	37 (29.8)	39 (34.8)		
Unemployed	5 (4.03)	3 (2.70)		
Father’s occupation			4.364^b^	0.111
Employer / Professional	84 (67.7)	67 (59.8)		
Employee / Non-professional	40 (32.3)	42 (37.5)		
Unemployed	0 (0.00)	3 (2.70)		
Family income			0.400	0.602
< 4000 RMB	58 (46.8)	57 (50.9)		
≥ 4000 RMB	66 (53.2)	55 (49.1)		

^a^ Children who had a sweet intake < once/day were treated as the low frequency of sweet intake group (LS group) and those with a sweet intake ≥ once/day were treated as the high frequency of sweet intake group (HS group).^b^by Fisher’s exact probability test

**Table 2:** Distribution of social behaviour factors between NC and S-ECC group^a^

Indicator	NC group (*n* = 118)	S-ECC group (*n* = 118)	*x*^2^	*P*
Gender			0.610	0.435
Boy	55 (46.6)	61 (51.7)		
Girl	63 (53.4)	57 (48.3)		
Single child			0.155	0.694
No	53 (44.9)	50 (42.4)		
Yes	65 (55.1)	68 (57.6)		
Caregiver’s type			3.401^b^	0.183
Parent	95 (80.5)	83 (70.3)		
Grandparent	22 (18.6)	34 (28.8)		
Other	1 (0.9)	1 (0.9)		
Marital status of parents			0.254^b^	0.614
Cohabiting	115 (97.5)	117 (99.2)		
Not Cohabiting	3 (2.5)	1 (0.8)		
Mother’s education			15.275	< 0.001
≤ 12 year	5 (4.2)	25 (21.2)		
>12 year	113 (95.8)	93 (78.8)		
Father’s education			1.967	0.161
≤ 12 year	7 (5.9)	13 (11.0)		
>12 year	111 (94.1)	105 (89.0)		
Mother’s occupation			0.711	0.701
Employer / Professional	79 (66.9)	73 (61.9)		
Employee / Non-professional	35 (29.7)	41 (34.7)		
Unemployed	4 (3.4)	4 (3.4)		
Father’s occupation			5.743^b^	0.057
Employer / Professional	81 (68.6)	70 (59.3)		
Employee / Non-professional	37 (31.4)	45 (38.1)		
Unemployed	0 (0.0)	3 (2.5)		
Family income			1.374	0.241
< 4000 RMB	62 (52.5)	53 (44.9)		
≥ 4000 RMB	56 (47.5)	65 (55.1)		
Frequency of sweet intake			6.797	0.009
< 1 time/day	72 (61.0)	52 (44.1)		
≥ 1 time/day	46 (39.0)	66 (55.9)		
Frequency of tooth brushing			0.434	0.51
≥ 1 time/day	105 (89.0)	108 (91.5)		
< 1 time/day	13 (11.0)	10 (8.5)		
Use of fluoride toothpaste			0.256	0.613
No	20 (16.9)	23 (19.5)		
Yes	98 (83.1)	95 (80.5)		

^a^S-ECC group were those children with decayed, missing, and filled teeth value equal to or more than six, and the non-caries (NC) group had no caries.^b^by Fisher’s exact probability test

### Genotype distribution of the rs35874116 polymorphism

In total, 187 (79.2%) children were TT genotype carriers, 46 (19.5%) were CT genotype carriers, and the remaining three (1.3%) were CC carriers. In subsequent analysis, the CT and CC genotype carriers were combined into one group (CT/CC carrier), as in a previous study [15].

The distribution of genotypes between sexes was not statistically significant (*x*^2^ = 0.0863，*p* = 0.769). Children of the TT genotype carrier consumed a higher frequency of sweets than CT and CC carriers (51.3% vs. 32.7%; *x*^2^ = 5.436, *p* = 0.020). There were more TT genotype carriers in the S-ECC group than in the NC group (53.5% vs. 46.5%; *x*^2^ = 4.353, *p* = 0.037). Distribution of the rs35874116 SNP between different genders, frequencies of sweet intake and caries status Table 3.

**Table 3:** Distribution of the rs35874116 SNP between different genders, frequencies of sweet intake and caries status

Variables	TT genotype	CT/CC genotype	*x*^2^	*P*
Gender				
Boy	91 (48.7)	25 (51.0)	0.086	0.769
Girl	96 (51.3)	24 (49.0)		
Frequencies of sweet intake			5.436	0.020
LS group	91 (48.7)	33 (67.3)		
HS group	96 (51.3)	16 (32.7)		
Caries status			4.353	0.037
NC group	87 (46.5)	18 (36.7)		
S-ECC group	100 (53.5)	31 (63.3)		

### Association of rs35874116 polymorphism with sweet intake frequency

The result of the relationship between rs35874116 polymorphism *with* sweet intake frequency in the full model by multiple logistic regression analysis was showed in Table 4. After adjusting for the child’s gender, single child, type of caregiver, parental marital status, educational level, and occupation, monthly family income, the TT genotype was significantly related to children’s frequency of sweet intake (OR = 2.25, 95% CI = 1.14–4.44).

**Table 4:** Summary of logistic regression analysis for rs35874116 polymorphism in relation to frequencies of sweet intake

Variables	ORs for frequencies of sweet intake (95% CI)
rs35874116 genotype	
Genotype CT / CC	1.00 (referent)
Genotype TT	2.33 (1.16–4.72)
Gender	
Boy	1.00 (referent)
Girl	0.64 (0.37–1.10)
Single child	
No	1 (referent)
Yes	0.99 (0.58–1.73)
Type of caregiver	
Parent	1.00 (referent)
Grandparent	1.06 (0.56–1.99)
Others	0.00 (0.00)
Marital status of parents	
Cohabiting	1.00 (referent)
Not Cohabiting	0.00 (0.00)
Mother’s education	
≥ 12 years	1.00 (referent)
< 12 years	0.86 (0.37–1.99)
Father’s education	
≥ 12 years	1.00 (referent)
< 12 years	1.17 (0.38–3.61)
Mother’s occupation	
Employer / Professional	1.00 (referent)
Employee / Non-professional	2.03 (0.44–9.46)
Unemployed	2.21 (0.47–10.41)
Father’s occupation	
Employer / Professional	1.00 (referent)
Employee / Non-professional	0.00 (0.00))
Unemployed	0.00 (0.00)
Monthly family income	
< 4000 RMB	1.00 (referent)
≥ 4000 RMB	0.76 (0.44–1.32)

### Association of rs35874116 polymorphism with S-ECC

The result of the relationship between rs35874116 polymorphism with S-ECC in the full model by multiple logistic regression analysis was showed in Table 5. After controlling for social behaviour factors such as gender, single child, type of caregiver, parental marital status, educational level, occupation, monthly family income, tooth brushing frequency, and use of fluoride toothpaste, the TT genotype was significantly related to S-ECC (OR = 2.11, 95% CI = 1.01–4.42). The frequency of sweet consumption was associated with high caries (OR = 1.82, 95% CI = 1.01–3.27). In addition, it was also found that the mother’s education level was also a significant contributing factor for the risk of S-ECC (OR = 7.17, 95% CI = 2.63–19.57).

**Table 5:** Summary of logistic regression analysis for rs35874116 polymorphism in relation to S-ECC

Variables	ORs for caries status (95% CI)
rs35874116 genotype	
Genotype CT / CC	1.00 (referent)
Genotype TT	2.17 (1.04–4.56)
Gender	
Boy	1.00 (referent)
Girl	0.78 (0.45–1.40)
Single child	
No	1.00 (referent)
Yes	1.25 (0.70–2.26)
Type of caregiver	
Parent	1.00 (referent)
Grandparent	2.01 (1.06–4.14)
Others	1.89 (0.08–40.76)
Marital status of parents	
Cohabiting	1.00 (referent)
Not Cohabiting	1.52 (1.09–2.11)
Mother’s education	
≥ 12 years	1.00 (referent)
< 12 years	10.18 (3.20–32.43)
Father’s education	
≥ 12 years	1.00 (referent)
< 12 years	0.77 (0.21–2.84)
Mother’s occupation	
Employer / Professional	1.00 (referent)
Employee / Non-professional	1.61 (0.28–9.08)
Unemployed	
Father’s occupation	
Employer/professional	1.00 (referent)
Employee/non-professional	0.00
Unemployed	0.00
Monthly family income	
< 4000 RMB	1.00 (referent)
≥ 4000 RMB	1.37 (0.77–2.45)
Frequency of sweet intake	
< once/day	1.00 (referent)
≥ once/day	1.83 (1.02–3.28)
Frequency of teeth brushing	
≥ 1 time/day	2.18 (0.77–6.17)
< 1 time/day	1.00 (referent)
Use of fluoride toothpaste	
Yes	1.00 (referent)
No	1.32 (0.61–2.84)

## Discussion

The development of caries is affected by a combination of environmental and genetic factors, bacterial factors, dietary factors, fluoride exposure, oral hygiene, saliva composition and flow rate, and tooth structure [23]. Sweet intake frequently is an important risk factor for caries, and sucrose in carbohydrates is believed to be the most cariogenic [24]. This study is the first to show evidence for the association of rs35874116 polymorphism with sweet intake as well as dental caries among Chinese preschool children. Our study demonstrated participants who were TT-type carriers were significantly prone to have a higher frequency of sweet consumption and suffer from S-ECC.

In most studies, the frequency of the TT type was 38.5–60.7% among Caucasians [9–15]. However, our study was conducted in a sample of Chinese individuals that had a higher frequency of TT type at 79.2%, which was similar to that found in a study in Korea (74.1%) [25]. Therefore, this may indicate that genotypes of rs35874116 in *TAS1R2* vary across ethnic groups, and the proportion of TT-type carriers was higher among Asian individuals than among Caucasian individuals. However, further investigation is required to establish the pattern of distribution of rs35874116 polymorphism in other ethnic groups. In addition, the distribution was not significantly different between sexes, which is similar to previous studies [9, 13], indicating that sex was not a determining factor impacting variants of rs35874116.

The present study provides evidence for gene variation of rs35874116 within *TAS1R2*, which is related to both sweet consumption frequency and dental caries. One plausible explanation may be that the rs35874116 polymorphism resides in the predicted first large extracellular domain of the T1R2 receptor, which hypothetically contains the ligand-binding site for carbohydrates and dipeptide sweeteners, and consequently impacts sugar-dependent disease [7, 26]. We found that carriers of the TT genotype of rs35874116 had higher frequency of sweet consumption, and this result in the current study is consistent with the results of two previous studies [8, 15]. However, this result in the current study is in contrast to the previous studies [13, 14], in which they found CC/CT carriers consumed more carbohydrates [13] or more sweet foods [14] than TT carriers. Moreover, our result is also inconsistent with the results of the other studies [10, 16], which showed that there was not any association between rs35874116 polymorphism and sweet intake [10, 16]. The same is true for the relationship between rs35874116 polymorphism and caries status. TT genotype carriers seemed to have a high risk of dental caries in permanent teeth in school children and adults [9, 11, 17]. Similarly, we found that S-ECC prevalence was higher among TT genotype carriers than CT / CC carriers, in contrast to a study in the Czech Republic, which showed that school children with the CT / CC carriers were more frequently affected by caries than children who were TT carriers [12]. The results varied in different studies, and these differences may be caused by ethnic differences between populations and gene–environment interactions, study design, mode of inheritance, and food culture.

The strength of the present study includes a relatively large sample size, which comprised 118 caries-free and 118 S-ECC children, whereas most other studies only examined a small group [8–12, 14, 15, 17]. In addition, multiple analysis was used to confirm the relationship between gene variation within rs35874116 and sweet intake as well as dental caries. The study results need to be interpreted with caution in light of several limitations. First, when we explored the relationship between gene polymorphism and sweet intake or caries, we just focuses on only one SNP locus in *TAS1R2* and didn’t consider other SNPs which might be connected with sweet intake or caries. Second, sweet intake was measured with a single, dichotomized question, and our future work should include other measures of sweet preference and/or do more to measure actual consumption of carbohydrates. Finally, the subjects included only Chinese preschool children. Thus, as sweet intake changes with age, the potential relevance of the findings to an older Chinese population will require future study.

## Conclusions

In conclusion, our study implies a possible influence rs35874116 polymorphism in *TAS1R2* on sweet intake and caries risk in Chinese preschool children. However, these findings require further confirmation in older Chinese populations. Understanding sweet intake and dental caries at the molecular level has the potential to facilitate new, more targeted approaches to the prevention and treatment of caries.

## Data Availability

All data generated or analysed during this study are included in this published article.
